# Experiences of Parents of Preterm Children Hospitalized Regarding Restrictions to Interact with Their Children Imposed Because of the COVID-19 Pandemic

**DOI:** 10.17533/udea.iee.v39n2e10

**Published:** 2021-06-15

**Authors:** Sandra Patricia Osorio Galeano, Ángela María Salazar Maya

**Affiliations:** 1 Nurse, M.Sc., Doctoral candidate. Professor. Facultad de Enfermería de la Universidad de Antioquia, Medellín (Colombia) Email: sandra.osorio@udea.edu.co Universidad de Antioquia Universidad de Antioquia Medellín Colombia sandra.osorio@udea.edu.co; 2 Nurse, Ph.D. Full Professor. Facultad de Enfermería de la Universidad de Antioquia, Medellín (Colombia) Email: angela.salazar@udea.edu.co Universidad de Antioquia Universidad de Antioquia Medellín Colombia angela.salazar@udea.edu.co

**Keywords:** pandemics, coronavirus infections, neonatal nursing, infant, premature, parents, intensive care units, neonatal, family nursing, pandemias, infecciones por coronavirus, enfermería neonatal, recién nacido prematuro, padres, unidades de cuidados intensivos neonatal, enfermería de la familia., pandemias, infecções por coronavirus, enfermagem neonatal, recém-nascido prematuro, pais, unidades de terapia intensiva neonatal, enfermagem familiar.

## Abstract

**Objective.:**

To describe the experiences of parents of hospitalized preterm children regarding the restrictions implemented in the neonatal intensive care unit -NICU- during the COVID-19 pandemic.

**Methods.:**

Qualitative study. Semi-structured interviews were conducted between April and October 2020 with 12 parents of preterm children, whose children were hospitalized and discharged from NICU during the pandemic. The analysis was performed with tools from grounded theory through open and axial coding.

**Results.:**

The study identified four categories regarding the experience: 1) needing information: refers to the need for clear and close information to compensate for the physical distance; 2) limiting the interaction with the children: expressed as a painful situation, which minimizes opportunities for learning to care at home for their preterm child; 3) the pandemic: adding to the fears: in which the virus appears as a new threat for the children, who are vulnerable given their premature condition; and 4) limiting social support after discharge: identifies that the parents had less family and professional support for care after discharge during times of pandemic.

**Conclusion.:**

Parents of preterm children lived a difficult experience that became complex within the context of the pandemic. The experiences of parents of preterm children during times of COVID-19 indicate that restrictions to enter neonatal units to prevent the virus transmission limited the interaction with the preterm child and with the health staff and increased the needs for information.

## Resumen

**Objetivo.:**

Describir las experiencias de padres de niños prematuros hospitalizados frente a las restricciones implementadas en la Unidad de Cuidado Intensivo Neonatal -UCIN- durante la pandemia por la COVID-19.

**Métodos.:**

Estudio cualitativo. Se realizaron entrevistas semiestructuradas entre abril y octubre de 2020 a 12 padres de niños prematuros; los niños estuvieron hospitalizados y dados de alta en UCIN durante la pandemia. El análisis se realizó con herramientas de la teoría fundamentada mediante codificación abierta y axial.

**Resultados.:**

Se identificaron cuatro categorías en torno a la experiencia: 1) Necesitando información: se refiere a la necesidad de información clara y cercana para compensar la distancia física; 2) limitando la interacción con los hijos: se expresa como una situación dolorosa, que minimiza las oportunidades de aprendizaje para el cuidado de su hijo prematuro en casa; 3) La pandemia: sumando a los miedos, en la que el virus aparece como una nueva amenaza para los hijos, quienes son vulnerables dada su condición de prematurez; y 4) Limitando el apoyo social después del alta: se identifica que los padres tuvieron menos apoyo familiar y profesional para el cuidado después del alta en tiempos de pandemia.

**Conclusión.:**

Los padres de los niños prematuros vivieron una experiencia difícil que se complejizó en el contexto de la pandemia. Sus experiencias, en tiempos de COVID 19, indican que las restricciones de ingreso a las unidades neonatales para prevenir la trasmisión del virus limitaron la interacción con el hijo prematuro y con el personal de salud e incrementaron las necesidades de información.

## Resumo

**Objetivo.:**

Descrever as experiências de pais de crianças prematuras hospitalizadas frente às restrições implantadas na Unidade de Terapia Intensiva Neonatal -UCIN- durante a pandemia do COVID-19.

**Métodos.:**

Estudo qualitativo. Entrevistas semiestruturadas foram realizadas entre abril e outubro de 2020 com 12 pais de crianças prematuras, cujos filhos foram hospitalizados e receberam alta da UTIN durante a pandemia. A análise foi realizada com ferramentas da teoria fundamentada por meio de codificação aberta e axial.

**Resultados.:**

Quatro categorias foram identificadas em torno da experiência: 1) Necessidade de informação: refere-se à necessidade de informações claras e próximas para compensar a distância física; 2) limitação da interação com os filhos: expressa-se como uma situação dolorosa, que minimiza as oportunidades de aprendizagem para cuidar do filho prematuro em casa; 3) A pandemia: agravando os medos: em que o vírus surge como uma nova ameaça às crianças, vulneráveis ​​pela prematuridade; e 4) Limitação do suporte social após a alta: identifica-se que os pais tiveram menos apoio familiar e profissional para o cuidado após a alta em momentos de pandemia.

**Conclusão.:**

Os pais das crianças prematuras tiveram uma experiência difícil que se tornou mais complexa no contexto da pandemia. As experiências de pais de bebês prematuros na época do COVID 19 indicam que as restrições de admissão em unidades neonatais para prevenir a transmissão do vírus limitaram a interação com o bebê prematuro e com o pessoal de saúde e aumentaram as necessidades de informação.

## Introduction

The COVID-19 pandemic, which demanded important adjustments in the health care of preterm children hospitalized in Neonatal Intensive Care Units (NICU), aimed at limiting exposure to the virus and preventing infection due to this cause. In general, children born prior to 37 weeks of gestation have greater predisposition to infectious diseases due to their immunological immaturity. Furthermore, although in the case of infection through the new coronavirus, this immaturity seems to avoid uncontrolled immune response and favor a favorable clinical course,(1) the need is without question to implement measures in neonatal units to protect preterm children and avoid their infection.

Infection through the new coronavirus has had a strong impact on the lives, health, and wellbeing of people throughout the world, where, according to data from the World Health Organization,(2) by 12 April 2021, COVID-19 had infected over 136-million people and caused nearly 2.9-million deaths, with the region of the Americas being the most affected. Colombia, by this same date, has reported over 2.5-million people infected and 65,889 people dead due to this cause.(2) This situation has led to entry restrictions to families to health institutions to avoid possibilities of infection and, particularly, in the NICUs, these restrictions limited possibilities of accompaniment, contact, and interaction by the parents with their children.

Although the incidence of COVID-19 in premature children is low and the expression of disease is predominantly slight,(3) it is necessary to implement strategies to prevent transmission to preterm children in NICUs, which also contributes to preventing the infection in the parents and the health staff in these care settings. The principal adjustments in neonatal care settings have had to do with restrictions for accompaniment by the parents of their children in neonatal units. These measures that, although necessary, generate great concern, given that it has been described that close companionship during hospitalization brings important benefits at physical and emotional levels for the parents and their children.(4-7) After years of promoting in Colombia the opening of the neonatal units to the parents and of working to favor their prolonged permanence during their children’s hospitalization, the COVID-19 pandemic, almost unexpectedly, demanded an opposing attitude, whose effects are yet unknown. Given the scarce information on the theme and considering the importance of knowing the experiences of the parents within this context, this article sought to describe the experiences of parents of preterm children hospitalized in the neonatal intensive care unit, regarding restrictions implemented during the COVID-19 pandemic.

## Methods

A qualitative study was conducted, using tools from grounded theory to analyze the data. This study is part of a broader research that seeks to describe the experiences of parents of preterm children regarding birth, hospitalization, and transition to the home. During the field work, the COVID-19 pandemic was declared, which permitted identifying the experiences of the parents with regards to the restrictions adopted in the neonatal unit, which were expressed cross-sectionally during the trajectory with their children and became a core of analysis, which identified categories that gained strength, growing in density and depth. Of the number of participants in the principal study, 12 parents lived the experience of having a preterm child hospitalized in an NICU during the COVID-19 pandemic; with this number, it was possible to identify properties and establish the saturation of the categories identified. None of the parents refused to participate in the study.

The inclusion criteria were: parents of children born before 37 weeks of gestation, whose children had been hospitalized in NICU, who were currently at home, and who had been discharged from the hospital not over three months. The study excluded parents whose children had some health problem at the time of the interview. Semi-structured interviews were conducted between April and October 2020, lasting between 30 and 80 minutes, through video calls or phone calls, given restrictions for social contact due to the pandemic. During the interviews, no other family member was present. A snowball convenience sampling was carried out, that is, a father or mother was contacted, which referenced another with the same experience and so forth, obtaining information related with study phenomenon.

The principal researcher established a telephone relationship with the participants to invite them to participate in the study and schedule the interview, at which time she introduced herself to the father or mother, indicating her credentials as nurse and nursing PhD student, in addition to informing about the objective of the study and the reasons to conduct the research, given her interests as a nurse from the area of neonatal care, in generating contributions to the quality of life of the parents and their preterm children. The interviews were carried out by the principal researcher who is a nurse specialized in neonatal nursing, Master’s and Nursing PhD student, with formation and research and professional experience in the area. The number of interviews was determined through theoretical saturation, understood as the point at which no new properties, dimensions or relationships emerge during the analysis and at which response is given to the study objectives.

The general study had a guide that was subjected to a pilot study to evaluate the pertinence and clarity of the questions in the context. During the research process, new questions emerged on par with the theoretical sampling, which permitted delving into some themes. The interviews were recorded and transcribed textually, within 48 hours after they were conducted; thereafter, this was returned to each participant via WhatsApp or E-mail, inviting them to make contributions or corrections according to what they considered. It was not necessary to conduct new interviews of the participants, given that the encompassed the themes proposed in the interview. The interaction with the participants permitted identifying emotions, reactions, and feelings that were registered in field notes.

The data analysis was carried out through line-by-line open encoding, identifying incidents to assign codes; this process was performed by the principal researcher and was contrasted with the analysis by another researcher in the study. An analysis matrix was carried out in which the codes were entered, giving way to the categories and subcategories. Diagrams were used as visual representations of the analytic scheme, which facilitated the interpretative process and identification of the points that needed development. Also, axial coding took place, which made the data comprehensible, by reducing its constitutive elements, relating the categories with their subcategories to form more complete descriptions through constant comparison of the data. No software was used for the analysis. 

Credibility, auditability, and transferability criteria were conserved; the entire process was crossed by the reflexivity of the researchers.(8) In function of the credibility, verifications were made during the interviews through oral synthesis or repetitions to ensure that the interpretations by the researcher were consistent with the declarations by the participants. In addition, the interviews were returned and keeping open the possibility of making contributions during the process. Auditability was achieved through the constant comparison of different views toward the object of study in the coding and categorization among the researchers. Transferability or applicability, as possibility of extending the study results to other populations will depend on how much the results fit another context; the readers will determine if they can transfer the findings to different contexts.

The study was supported by the research ethics committee in the Faculty of Nursing at Universidad de Antioquia minute # CEI-FE 2020-02. Informed consent was read and audio recorded, given that it was not possible to have face-to-face contact with the participants due to restrictions from the pandemic.

## Results

Twelve parents participated who had their children hospitalized in NICU during the pandemic, in different health institutions. The principal characteristics of the participants are shown in Table 1.

**Table 1:** Characterization of parents participating in the study and of their children

Information about the parents	Information about the parents	Information about the parents	Information about the parents	Information about the parents	Information about the children	Information about the children	Information about the children	Information about the children	Information about the children
Code	Sex	Age	Schooling	Residence	Weeks of gestation	Multiple birth	Weight at birth in grams	Days of hospital stay	Days after discharge
P1	F	29	University	Urban	28	No	1180	83	15
P2	F	20	University student	Urban	28	No	1005	48	39
P3	F	24	University	Urban	34	No	1830	39	32
P4	M	34	Complete primary	Urban	34	No	1830	39	32
P5	F	29	Complete secondary	Urban	31	No	1420	32	15
P6	F	35	Incomplete secondary	Urban	34	No	2135	16	90
P7	F	30	Technical	Urban	26	No	900	69	120
P8	F	32	Technical	Rural	32	Yes	Son 1: 1920	48	44
P8	F	32	Technical	Rural	32	Yes	Son 2: 1580	48	44
P9	M	52	Complete secondary	Rural	31	No	1775	34	15
P10	F	35	University	Urban	30	No	1056	59	30
P11	F	25	Complete secondary	Urban	26	No	840	60	28
P12	M	25	Technical	Urban	32	Yes	Daughter 1: 1610	Daughter 1: 17	Daughter 1: 29
P12	M	25	Technical	Urban	32	Yes	Daughter 2: 1614	Daughter 2: 19	Daughter 2: 31
P12	M	25	Technical	Urban	32	Yes	Daughter 3: 1200	Daughter 3: 34	Daughter 3: 1

* At the time of the interview

Amid the diversity of the social characteristics of the participants, common experiences were found that broadened comprehension of the phenomenon. The results confirm that the experience of having a preterm child is complex for the parents and which is much more within the context of the pandemic. The following categories emerged: *1) Needing information, 2) limiting the interaction with the child 3), the pandemic: adding to the fears and 4) limiting social support after discharge*.

### First category. Needing information

Preterm birth and hospitalization in the neonatal unit are critical and unexpected events for the parents, which are removed from the ideal imagined about their child’s birth. Uncertainty is common in parents and information is one of the greatest needs during the experience. The category needing information has as subcategories: “needing general information about the child” and “needing information about the pandemic”.

The subcategory **needing general information about the child** reveals - in the first place - those parents need more than ever close and constant information because they cannot accompany permanently their children due to entry restrictions. The measures adopted varied from one institution to another and in all cases limited the time of their remaining in the unit, thus, increasing the needs for information. In this regard, the parents stated: *There was much restriction, it was only one hour in the morning and one hour in the afternoon* (P08). *Because of the pandemic, it was by beds, one day was for the mothers of children in even-numbered beds and the next for those in odd-numbered beds* (P12). Under these circumstances, the parents received information via telephone and WhatsApp that, although not replacing the need to be near their child, helped them and gave them relief. With greater information, the parents felt better with respect to the situation. One account indicated: *With the pandemic, every day they sent me a report on the status, they reported via WhatsApp; that information was like the daily bread, like the day’s most important meal, like the day’s biggest blessing* (P10).

Information from the health staff about the status of their children gained special sense during times of pandemic. The parents described the need for information as urgent because it was a way of connecting with their children’s reality, which permitted bridging the painful physical distance. This general information the parents needed had to do with the status of their children, progress in the process, treatments and other aspects related with their care. But the parents indicated needing detailed, close, and personalized information; given that in some cases they identified that the information did not respond to their expectations: *When calling, the information was very mechanical; they would tell me: they are calm, ate well, are well, are asleep. I know it by memory and because you don’t have the tools to know if that was so, then you said thank you, they are in your hands (*P12).

The pandemic required creating additional communication channels to keep the parents abreast of the daily evolution of their children. This is how the nursing staff resorted to alternatives, like photographs, which were appreciated positively by the parents. In this respect, one of the mothers expressed: *in the photograph from the first day the she had a bunch of devices, I was overcome by seeing her with so many things, I worried; but little by little, in the pictures, I started seeing they were removing the implements she had attached and it gave me come calm.* (P10). Moreover, the subcategory **needing information about the pandemic** indicates that besides information about their children, the parents need to know about the pandemic and particularly about the reasons for the restrictions. Given the adequate information about the reasons for limiting their entry, the parents accepted and appreciated these types of controls: *They explained to us what they were doing, it was for the good of the babies, to care for them, they tested me twice, as a requirement* (P11).

The parents stated that separating from their children was painful and difficult to assume, but information gave them some tranquility and helped them to accept the situation: *They would explain that is was to care for the children. I hurt you, but when they explained it that way, you accepted it and calmed down* (P10). Parents of preterm children during times of pandemic need clear, close, and constant information from the health staff about their children, but also need to be guided about the need to limit their entry and contact with their children, to facilitate accepting the circumstances. Video calls, daily telephone reports, photographs and videos were of great importance for the parents.

### Second category. Limiting the interaction with the children

In general, parents of hospitalized preterm children had the need to be near their children. Even in the intensive care setting, when the children’s physiological instability did not permit direct interaction, the parents felt relief when seeing their children in an incubator and knowing first-hand the events related with their health. During the pandemic, limitations for the interaction were expressed in two subcategories: “living the physical separation” and “limiting opportunities of preparation for caring”.

Living the physical separation is a subcategory that indicates that the closeness of the parents with their children in the neonatal unit is an emotional need, which is why the separation is a painful event. The start of the quarantine increased the emotional burden, pain, and stress of the parents. On this theme, some testimonies were: *that weekend of the quarantine that we could not go to see her, oh, I cried and was very stressed! Knowing it would be three days without seeing her, you can’t imagine what one feels!* (P5). *There were two days that I did not go, the weekend when the quarantine started; for me, it was the longest weekend in history* (P2). During the quarantine, interaction was limited and although they got daily information, seeing their children from afar had for them great value: *I could see her from far away, through the glass I managed to see how she was and through the glass I would say hi and tell her: my princess, I am here to leave you the milk and that I loved her a lot* (P10).

The neonatal unit favors contact and active participation in the care when the child’s conditions so permit. Direct interaction by the fathers with their children permits their developing their paternal role, receiving support and direct information from the health staff, and learning about caring for their child. In some units, the adjustments included restricting the father’s entrance, only the mothers entered with assignment of days and schedules. Upon not having possibilities to accompany directly their children, the fathers were active through phone calls. One father expressed: *The role I assumed was that of staying in telephone contact with them, given that I could not be there, so I kept working, but calling in the morning and at night* (P12).

Except for the pandemic, paternal participation is promoted in all the care activities within the unit, but in spite of their disposition and interest, the reality of COVID-19 annulled - in some cases - the possibilities of participation by the fathers during hospitalization, permitting entry only to the mothers. In other cases, although the fathers could enter the unit with restricted schedules, they experienced logistic limitations to be with their children and only the mothers assumed this companionship. Some testimonies in this regard were: *we come from a village, with that of the pandemic and work, I would tell him: why are you going to come if you can only stay for only one hour* (P7)*. Given that he had to work, he had to return and I stayed alone, he saw her again when we arrived here* (P10). This aspect bears special relevance because mutual support is of great importance within the process and it is expected that both parents receive information and develop skills to care for their child.

The subcategory **limiting the opportunities to learn about caring for the premature child** indicates that because of the limited permanence in the neonatal unit, the fathers had less opportunities to learn and develop confidence to care for their child. The care preterm children receive at home is determinant for their health and wellbeing, due to that, the skills of the parents for caring is a criterion for discharge. Within the context of the pandemic, the preparation for the discharge was carried out in the settings of basic care, when the discharge was imminent, in contrast with the habitual conditions in which the parents tend to initiate the preparation process since the admission. The fathers recognize that with greater accompaniment time there are greater opportunities to gain confidence for care: *I went some times and paid attention to see how to handle him, I managed to practice two times* (P9)*. Before giving her to me, she was in basic care and those last days they allowed me to enter, the nurses guided me; but as a mother you must be there more, so that when you leave, you leave secure* (P10). The processes of accompaniment are fundamental in aspects as sensitive as breastfeeding. This is a process in which the mothers need a lot of support from the nursing staff, given that it is a complex experience due to prematurity of their children. The pandemic also generated limitations at this level: *I got her to breastfeed two days before the lockdown began and then I could not breastfeed her* (P5).

One of the specific cares of preterm children is skin-to-skin contact through the kangaroo care method. This process starts during the hospitalization and, upon discharge, it is expected that both parents have gained skills and confidence. Due to the restrictions, some fathers did not have the possibility of “kangarooing” (carrying the premature child in vertical position between the mother’s breasts or the father’s chest with the face lateralized and the upper and lower limbs in frog position, guaranteeing direct skin-to-skin contact), during hospitalization: *The kangaroo care was after they gave him to me and got out of there; there, I could not kangaroo care* (P09). One of the biggest immediate effects of the pandemic in parents of preterm children, was the limited interaction with their fathers. This aspect causes great concern because contact and permanence of the fathers with their children is a condition that favors the development of skills for caring in the fathers and the paternal bond, aspects that are determinant in the continuity of the care of the children.

### Third category. The pandemic: adding to the fears

The parents of preterm children experienced fears related with the risks of the immaturity of their children and with the uncertainty regarding their evolution and survival. This is how the parents who participated in the study described an experience burdened with fears, in which the pandemic was one more of these. Fears due to COVID 19 were expressed in two subcategories: “fear of infection” and “fear of going home with their child amid the pandemic”. **Fear of infection** is added to the emotional burden and to the fears of the parents. The COVID- 19 pandemic appears as a new possible cause of death for their children: *The pandemic is another thing that stirs a lot of fear, the fear was also that, losing one of the babies* (P8).

Seeing their children is the biggest craving for the parents, but the fear of being a possible source of infection confronted them with a painful experience*.* One mother indicated: *this was very scary, you being in a clinic and having to go to your baby with the fear that perhaps you are bringing the virus, it is very hard* (P11). The parents confronted themselves regarding the risks of accompanying their children and the fear of infecting them confronted them with a personal dilemma: *It places you between a rock and a hard place because you have to take a risk and go out to the street without knowing if you will be infected and if you do you go and see your child and even if you wash your hands and where protective clothing, you are exposing the child too much* (P1).

The subcategory **fear of going home amid the pandemic** has to do in the first place with the recognition the parents have with respect to the importance of the follow up of their children after discharge, which is why they fear the pandemic will impede adequate follow up: *I was afraid because the kangaroo plan was closed because of the pandemic, so I was worried about the check up and the daily weight monitoring* (P10). Additionally, fears emerged about the daily life imposed by the quarantine. The parents recognize the possibility of negative health events of their children once they are at home and fear not being able to consult in timely manner: *my fear was that during the quarantine there was no transportation, I would go out and would see no taxis, or buses or anything. Then, I would say, I know I have to rush to the hospital if this or that happens, but if something does happen, what can I do if I have no transportation available* (P7).

The discharge tends to be a moment desired by the parents because it represents the closure of the most-critical phase of the process, but it was accompanied by the fear of infection of the virus, as shown by the testimony: *I felt joy and at the same time not too happy by this pandemic situation because going from here to there to take them to medical check-ups, you are risking yourself and the baby, that worried me* (P9). In contrast, for some parents, the discharge during the pandemic represented a relief because the situation added too much stress to the hospitalization process. *The discharge was a chance to rest, this virus, this contingency and this lockdown, but I was already home with my son* (P5).

The pandemic was an additional factor that added to the difficult experience the parents lived, as they so stated: *There were many things, this quarantine, I separated from the other child from whom I had not been separated for so long; it was too many things, it is very hard* (P08)*.* Fear was a common feeling in the parents during the pandemic, which accentuated the complexity of the experience, where a high emotional burden exists related with fears due to the vulnerability of their children.

### Fourth category. Limiting support after discharge

At home, the parents acquired new dynamics and made adjustments for the care of their children. Limitations for the follow up of their children and for the support from relatives or people close to them to care for the child were aspects generated due to the pandemic and are identified in the subcategories “limiting the follow up” and” limiting social support after discharge”. In the subcategory **limiting the follow up**, it is important to consider that after discharge, the parents tend to attend the kangaroo program to monitor their children according to their specific needs, which permits - among other aspects - assessing and enhancing their skills for care. This program is of vital importance to guarantee the wellbeing of preterm children.

The contingency due to the pandemic generated changes in the program by making the experience more complex: *due to the quarantine, the kangaroo plan has been via telephone, the pediatrician came the first two weeks and now it is once a week via telephone, everything has been quite complex* (P1). *I did not have a kangaroo plan, it was the kangaroo method we practiced here, as they had taught us* (P8). *Due to the pandemic, I could not be seen in the kangaroo plan, so the pediatrician sees her every month* (P10). In other cases, the fathers and their children did attend the kangaroo plan, but also faced entry restrictions. Only the mother could go in; the father could only enter to receive the medical information: *I was in the kangaroo plan only when the pediatrician was talking, but not during breastfeeding and the exercises because of the pandemic* (P12). In spite of the restrictions, the participants expressed their interest in active paternal participation in the kangaroo program: *I would have liked for the father to enter because he is very interested in his baby and wanted to know everything* (P11). For some fathers, who could not accompany their children during the care process, receiving direct information was an opportunity they appreciate and are grateful for, reaffirming the importance of personal interaction with the health staff and the need for information: *In the kangaroo plan, they said: dad, you cannot enter, I said: I want to know. The pediatrician finally let me enter and fortunately explained many things and I thank him for that* (P12).

Regarding the subcategory **limiting social support after discharge**, it is important to recognize that in the transition to the home the parents face a difficult reality, given that caring for a preterm child is highly demanding. Parents need help from people nearby, but the pandemic limited this possibility. Fear of infection appears as a threat to their children. Restricting the entrance to outside people was part of the measures adopted to protect their children at home: *When we got home, completely isolated, with nobody seeing or touching the baby, because we knew she could get sick* (P4)*.* Preterm children must be protected from possible respiratory infections, but within the COVID-19 context, recommendations in this sense increased, while increasing fears in the parents: *We would not let anybody come over; we stayed in the house because of the pandemic* (P10).

Limiting contact also reduced the possibilities for help. The pandemic and the fears it generated in the parents diminished the openness and disposition to receive support. Some testimonies were: with *the pandemic you are afraid of possibly infecting them, so no, nobody is going to come here (*P16)*. One day, an aunt offered to help me, but I told her no, that I was very afraid because you don’t know what symptoms she may have* (P11). The parents limited their going out, reportedly only going to appointments related with their children’s health, to avoid exposing them: *we do not go out for anything, the only one who goes out is the father to work* (P1). Some parents reported receiving telephone support from their families: *the support was mostly from my mother-in-law via telephone because - due to the pandemic - we are not receiving anybody* (P10). The parents who received support, got it from very close individuals: *My sister, she was with me, she would help me* (P03). *My mother always helped me* (P05)*.*

The risk of prematurity does not end with the hospitalization and the challenges the parents experience after the discharge are quite complex. The transition to the home supposes important challenges, which is why limitations in the follow up and in social support become a concern, whose effects in the children and their parents are still unknown.

## Discussion

Prevention of COVID-19 infection in neonatal units is a priority and amid the measures adopted with this purpose, restrictions against physical contact marked the experience of the parents; the effects of these measures upon the health and wellbeing of the children and their parents in the short and long term are still unknown. The birth of a preterm child, their hospitalization in the neonatal unit, and the transition to the home are complex phenomena in which the parents face a hard reality, burdened with pain, uncertainty, fear, anxiety, stress and guilt.(9,10) The complexities of these phenomena were accentuated during times of pandemic. The parents experienced a marked need for information, which also has been described in studies that have inquired about the needs of the parents of preterm children.(10,11)

It is fitting to recognize that, in the specific case of the parents interviewed, the need for information went beyond, converting it into a form of connection with the reality of their children. Besides information about their children, the parents need support, accompaniment, and a channel to solve their concerns. Virtual media permit human interaction, where it is possible to respond to these needs. It has been identified that strategies involving the use of information and communication technologies are useful in this purpose.(12) Furthermore, it has been documented that telephone support can help to solve their concerns in the process(13) and, with such, becomes an opportunity to strengthen preparation processes for the discharge.

Separation of the preterm children and their parents has negative effects at the physical and emotional levels and in the mental health of the parents and children.(14) The presence of the parents facilitates to the nursing staff the direct transference of information about the child’s care, but also permits establishing a relationship of trust,(15) provide support, and identify the needs and possibilities of each parent to assume effectively the care of their children, which is determinant for the child’s health after discharge. Due to this, the virtual media used during the pandemic must be a means of emotional support and an educational setting that complements the limited spaces that allow the physical presence of the parents, which becomes a challenge for the nursing staff.

During the pandemic, personal interaction was limited and telephone information, videos, and virtual resources, in spite of their usefulness within this context, do not substitute the personal interaction. Although efforts in this sense were of great importance and the health staff created possible communication channels amongst the circumstances, it is currently necessary to integrate theory and evidence(16,17) to guarantee involvement by the family, humanized care, and successful transition to the home amid the current possibilities.

One of the frameworks that must guide the strategies de accompaniment and support for parents of preterm children during the pandemic is that of family-centered care. This model, broadly disseminated globally and whose principles are applied in the different contexts of neonatal care, promotes the implication of parents in caring for their children, favors family bonding and empowerment to achieve more humanized care.(7) Although the pandemic supposes new logics in the interaction by both parents with their children, it is important to consider that these principles continue tracing the path of caring for preterm children, given that evidence exists of the positive health effects and wellbeing of preterm children and their families.(15) In this care focus, it is necessary to recognize that the presence of both parents is fundamental. Within this atypical context, it is urgent to consider that the adjustments made in the neonatal units cannot exclude the fathers; by doing so, it generates a reversal whose impact upon the child’s health and satisfaction by the fathers may be considerable. Limited interaction by the fathers with their children impacts negatively on their skills for care at home, which is why it is necessary - even during times of pandemic - to favor the male co-responsibility and promote the effective participation of the men in the paternity.(18)

Given that, currently, the physical presence by both parents in the neonatal unit is limited because of COVID-19, it is necessary to consider the urgency to compensate this distance through alternative communication means, like phone calls and video calls, not only as ways of transmitting information about their child’s health status, but also as a possible means to establish a therapeutic relationship that can provide care to aid and support and in which each father and mother can be assessed to start nursing interventions according with their specific needs, in function of their wellbeing and that of their children. These means also permit evaluating and potentiating social and family support networks to guide them toward the possibility of receiving help from significant persons, conserving the security and protection measures required by the current circumstances. 

Moreover, today more than ever, it is necessary to strengthen the application of disciplinary theories that guide the care of preterm children and their families in phenomena as complex as, for example, development of the paternal role, the parental link and bond, adaptation, the transition by the parents of preterm children, and social support. Knowledge by nursing and its integration to the practice permits responding to the challenges imposed by the times of pandemic.

In turn, the disciplinary theory must also guide research amid the pandemic, during which new and complex questions emerge in the different care scenarios.(17) The results of the present study reaffirm that virtual media have become a valid, possible, and safe strategy during times of pandemic, which complement face-to-face activities, which, although limited by the need to prevent COVID-19 infection, are necessary and irreplaceable. The fathers need the interaction with their children to develop skills to care at home, which is a multidimensional phenomenon that not only has to do with knowledge and abilities, but also with confidence, social support, the bond, and development of the parental role, aspects that cannot be ignored amid the contingency and which - on the contrary - must be re-dimensioned under these circumstances, which has been described as one of the biggest challenges for the health staff in the neonatal units_._(19)

## Conclusion

The experiences of parents of preterm children during times of the COVID-19 pandemic indicate that entry restrictions to neonatal units to prevent the virus transmission limited interaction with the preterm child and with the health staff and increased the needs for information. After discharge, the pandemic diminished the opportunities of follow up and social support. All these aspects are sensitive in the experience of parents of preterm children and the effects of these restrictions are still unknown, which is why it is necessary to conduct studies that permit knowing their effects on the health and development of the children, as well as on the wellbeing of the parents.

The study recommends guiding strategies to accompany preterm children, considering the principles of development-centered care involving both parents. Virtual media must complement and optimize live settings, which cannot be substituted and are necessary particularly in the development of skills for caring of preterm children at home.
